# Antixenosis in *Glycine max* (L.) Merr against *Acyrthosiphon pisum* (Harris)

**DOI:** 10.1038/s41598-021-94703-6

**Published:** 2021-07-27

**Authors:** Katarzyna Stec, Bożena Kordan, Iwona Sergiel, Magdalena Biesaga, Joanna Mroczek, Jan Bocianowski, Beata Gabryś

**Affiliations:** 1 Department of Botany and Ecology, University of Zielona Góra, Szafrana 1, 65-516 Zielona Góra, Poland; 2 Department of Entomology, Phytopathology and Molecular Diagnostics, University of Warmia and Mazury, Prawocheńskiego 17, 10-720 Olsztyn, Poland; 3 Department of Biotechnology, University of Zielona Góra, Szafrana 1, 65-516 Zielona Góra, Poland; 4 Department of Chemistry, University of Warsaw, Pasteura 1, 02-093 Warsaw, Poland; 5 Department of Mathematical and Statistical Methods, Poznań University of Life Sciences, Wojska Polskiego 28, 60-637 Poznań, Poland

**Keywords:** Chemical biology, Ecology, Ecology

## Abstract

To reveal the antixenosis potential against the pea aphid *Acyrthosiphon pisum* (Harris) (Hemiptera: Aphididae) we analyzed the pea aphid survival and probing behavior, and the quantitative and qualitative variation of flavonoids in the leaves of selected soybean *Glycine max* (L.) Merr (Fabaceae) cultivars ‘Aldana’, ‘Annushka’, ‘Augusta’, ‘Madlen’, ‘Mavka’, ‘Simona’, ‘Violetta’, and ‘Viorica’. Aphid survival was drastically impeded on all cultivars. The electronic monitoring of aphid probing using the Electrical Penetration Graph (EPG) technique revealed that on all soybean cultivars, *A. pisum* readily probed into leaf tissues but the probes were usually terminated before reaching vascular tissues, which demonstrates the activity of antixenosis mechanisms in peripheral tissues epidermis and/or mesophyll in soybean leaves. The potency of antixenosis factors differed among soybean cultivars, which was reflected in differences in aphid survival and frequency and duration of phloem sap ingestion. Seven flavonoids were found: apigenin, daidzein, genistein, glycitein, isorhamnetin, kaempferol, and rutin, which occurred in different amount and proportion in individual cultivars. The content of apigenin and genistein in all soybean cultivars studied probably made them relatively unacceptable to *A. pisum*. Kaempferol in ‘Aldana’ might be responsible for the observed strong antixenosis resistance of this cultivar to *A. pisum.* The results of our survey provide the first detailed data that can be used for future studies.

## Introduction

Soybean *Glycine max* (L.) Merr. (Fabaceae) is one of the most important world crops in both the temperate and tropical regions^1^. In 2019, the world production was over 349.4 million tons from the area of over 128.9 million ha and is still increasing^2^. In Poland alone, the acreage of soybeans cultivation increased from nearly 0.0 ha in 2015 to 7920 ha in 2019^2^. The growing demand for soybean derives from its multiple uses for human and animal consumption due to high content of protein and oil, industrial application such as biodiesel, and as a nitrogen-fixing ground cover^1^. Soybean is also a source of biologically active substances for medicinal application. The anti-microbial, anti-inflammatory, antioxidant, and anti-tumor activities of soybean flavonoids and saponins are broadly known^3,4^.

Within a guild of herbivores associated with soybeans, aphids (Hemiptera: Aphididae) have a special status. Aphids affect plant condition directly due to the removal of nutrients and are able to transmit plant pathogenic viruses. Two legume-associated aphid species: the soybean aphid *Aphis glycines* Matsumura and the pea aphid *Acyrthosiphon pisum* (Harris) are crucial in the transmission of major destructive viral pathogens in soybean production worldwide, including the non-persistent transmission of *Soybean mosaic virus* (SMV) and the persistent transmission of *Soybean dwarf virus* (SbDV)^5,6^. *A. glycines* has gained considerable attention of researchers in the recent years due to its mass occurrence, role in virus transmission, and expansion to all regions of soybeans cultivation^7–9^. The resistance potential in soybeans against *A. glycines* has been explored^9–11^ and the role of flavonoids in *A. glycines*—soybean interaction has also been studied in detail^12,13^. The relationship between *G. max* and *A. pisum* remains largely unknown, although the pea aphid occurs abundantly in ecosystems in the vicinity of soybean where its populations are supported by various wild legumes. It must be kept in mind that *A. pisum* is considered one of the most important pest insects of leguminous plants worldwide and it is able to transmit over 40 plant pathogenic viruses among these plants^14–17^. The pea aphid is also a vector of bacterial pathogens such as *Pseudomonas syringae*, which are excreted in the aphid honeydew^18^.

The pea aphid comprises at least 11 biotypes, each of which shows a different preference and performs differently on specific host plants^19^. On less preferred hosts, a given biotype of *A. pisum* shows a limited ability to ingest sap and reproduce, which may be due to the variation in plant characteristics depending on plant taxonomical position^20–23^. At the same time, we demonstrated that the pea aphids of *Pisum sativum*-derived biotype are able to successfully infest and feed upon various forage and grain legumes that are not the key host plants of this aphid biotype^24–26^. Due to the specificity of behavioral phases in the host-plant selection process, the pea aphid poses a potential threat as a virus vector not only to its optimal host plant but also to second-choice plants or non-hosts. During brief intracellular probes in epidermis and parenchyma that are essential for the recognition of host plants^27^, aphids transmit non-persistent and semi-persistent viruses and during probing in sieve elements, persistent viruses are transmitted^28,29^. On highly susceptible plant species or cultivars, the pea aphid probing and feeding activities are not impeded. On moderately susceptible plants, aphids have difficulty to attain the feeding phase. Finally, on resistant plants, the probing time is shortened, non-probing intervals between probes are long, and the success rate in reaching the feeding phase is very low or none^24–26^. A susceptible cultivar may become a reservoir for *A. pisum* population in agroecosystems and increase the risk of virus spread due to aphid infestation. Thus, the reduction in the duration of stylet penetration and especially the prevention of stylet penetration beyond the epidermis and mesophyll may lower the direct impact of the pea aphid on the yield and contribute to the decrease in the transmission of semi-persistent and persistent viruses^28^. These goals are often achieved in cultivars that exhibit antixenosis resistance. Antixenosis occurs when plant morphological or chemical factors adversely affect arthropod behavior, leading to delayed acceptance or rejection of a plant as a host^30–32^. Aphid resistance is often significantly correlated with levels of allelochemical antixenosis factors that may act as repellents or feeding deterrents and affect aphid behavior at different phases of stylet penetration activities^23,27,28,33–35^. Flavonoids, which occur abundantly in the soybean, are well-known mediators of plant–insect interactions and represent the major line of constitutive and induced defense against herbivory^36^. An increase in flavonoid contents has been observed in plants infested by *A. pisum*^37–39^. Flavonoids are synthesized in the cytosol and stored in vacuoles and apoplast or transported to other tissues via cell-to-cell movement or by phloem vessels^40–43^. This means that aphids may come into contact with flavonoids at various stages of stylet penetration, during brief cell punctures in epidermis and mesophyll and during sap ingestion from sieve elements.

In the course of our previous studies on the susceptibility of various species of grain legumes to *A. pisum*, we discovered strong antixenosis potential against the *P. sativum*-derived biotype of the pea aphid in soybean cv. ‘Aldana’, which was manifested in a shortened aphid stylet penetration time, long non-probing intervals between probes, and a very low success rate in reaching phloem elements as compared to the most preferred host plants *P. sativum* and *V. faba*^26^. Although *G. max* is not a favored host for the pea-biotype of the pea aphid, a relatively susceptible soybean cultivar may become a residue for this biotype in the agroecosystem, which may contribute to the risk of virus spread. Significant intraspecific variation in susceptibility to the pea-biotype of *A. pisum* occurs in scarlet runner bean *Phaseolus coccineus* L. and string bean *P. vulgaris* L.; these two species comprise cultivars that are highly resistant and relatively susceptible to *A. pisum*^26^. Variation in antibiosis and antixenosis towards *A. glycines* was also reported among various genotypes of *G. max*^11^.

The aim of the present study was to explore the antixenosis potential in a selection of *G. max* cultivars. We hypothesized that soybean cultivars from different regions of origin in Europe differ in the level of antixenosis towards the pea aphid and that these differences are linked to the content of flavonoids. We monitored the pea aphid choices of soybean cultivars, the ability to survive on these cultivars, and aphid stylet penetration activities in plant tissues. We applied the Electrical Penetration Graph (EPG, known also as electropenetrography) technique, which is crucial in determining the influence of antixenosis factors on individual phases of aphid probing in peripheral as well as in vascular plant tissues^23,27,28^. In addition, we analyzed the quantitative and qualitative variation in the content of major flavonoids in the leaves of the soybean cultivars.

## Results

### Free-choice test

**Figure 1:**
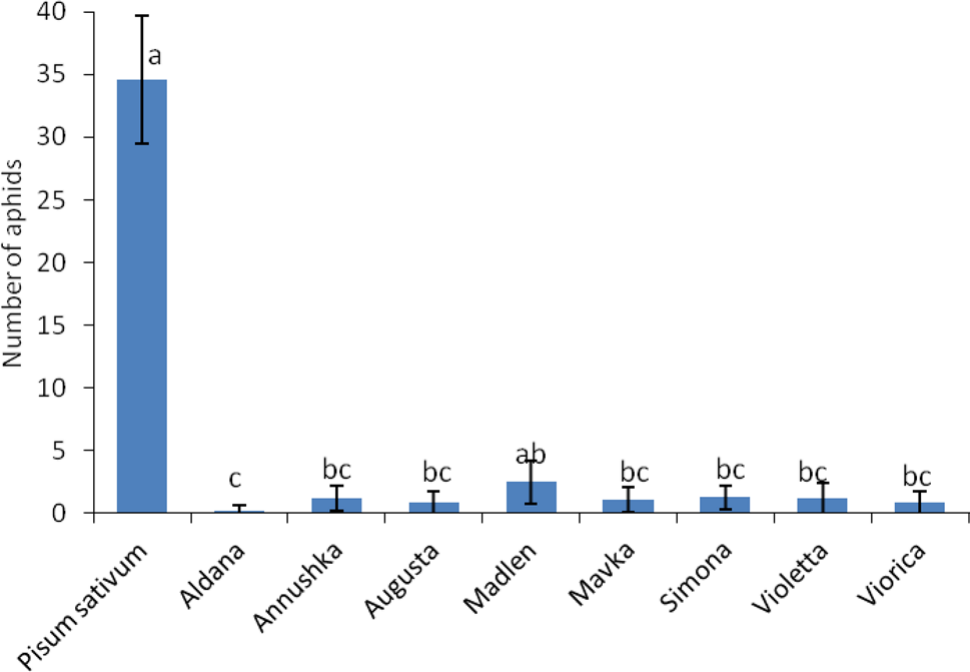
Number of *Acyrthosiphon pisum* apterous females (mean ± SD) in free-choice tests for soybean cultivars at 24 h after aphid introduction. For each test (n = 15), 50 *A. pisum* were introduced initially at the center of the Petri dish containing leaves of soybean cultivars arranged in a circle. *Pisum sativum* cv. ‘Milwa’ was used as susceptible check for all soybean cultivars. Bars followed by the different letters are significantly different (LSD_0.05_: 1.759; ANOVA *F*: 318.85).

Free-choice test was performed to study the variation in antixenosis-based resistance in soybean cultivars towards the pea-associated biotype of *A. pisum* (defined as ‘biotype ‘G’^19^). Twenty-four hours after aphid introduction, each of the eight tested soybean cultivars had statistically fewer aphids compared to susceptible control, *P. sativum.* At the same time, significantly fewer aphids chose soybean cv. ‘Aldana’ than the seven other cultivars, but no significant differences in the number of aphids were recorded among the other soybean cultivars (Fig. 1).

### Survival of the pea aphid on soybean cultivars

**Figure 2:**
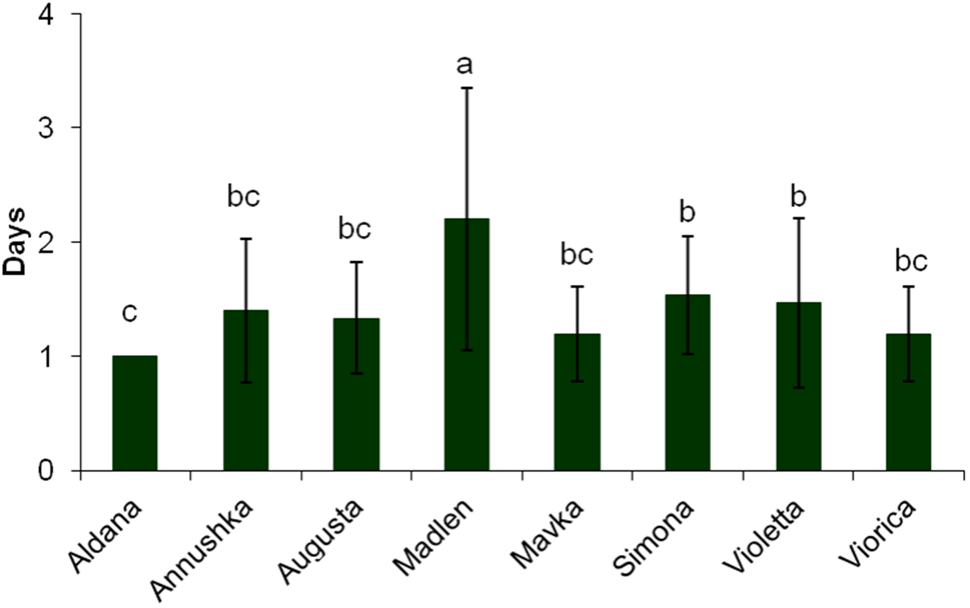
Survival of 1st instar nymphs of *Acyrthosiphon pisum* on *Glycine max* cultivars (n = 15; mean ± SD). Bars followed by the different letters are significantly different (LSD_0.05_: 0.451; ANOVA *F*: 4.98).

We applied a standard procedure to study aphid life parameters on soybean cultivars, in which an apterous female is transferred from the stock colony to the experimental plant, then the development of the first newborn nymph is monitored until its death as an adult. We recorded that, as expected, all apterous females transferred from peas gave birth to the nymphs. However, no 1st instar nymph molted into 2nd instar on any soybean cultivar. The average survival of 1^st^ instar nymphs of the pea aphid ranged from 1.0 (± 0.0) day on cv. ‘Aldana’ to 2.2 (± 1.4) days on cv. ‘Madlen’. Survival on ‘Simona’ and ‘Violetta’ was 1.5 (± 0.5) and 1.5 (± 0.7) days, respectively, and differed significantly from ‘Madlen’ but was similar to the survival on ‘Aldana’, ‘Annushka’, ‘Augusta’, ‘Mavka’, and ‘Viorica’ (Fig. 2).

### Probing behavior of the pea aphid on soybean cultivars

**Figure 3:**
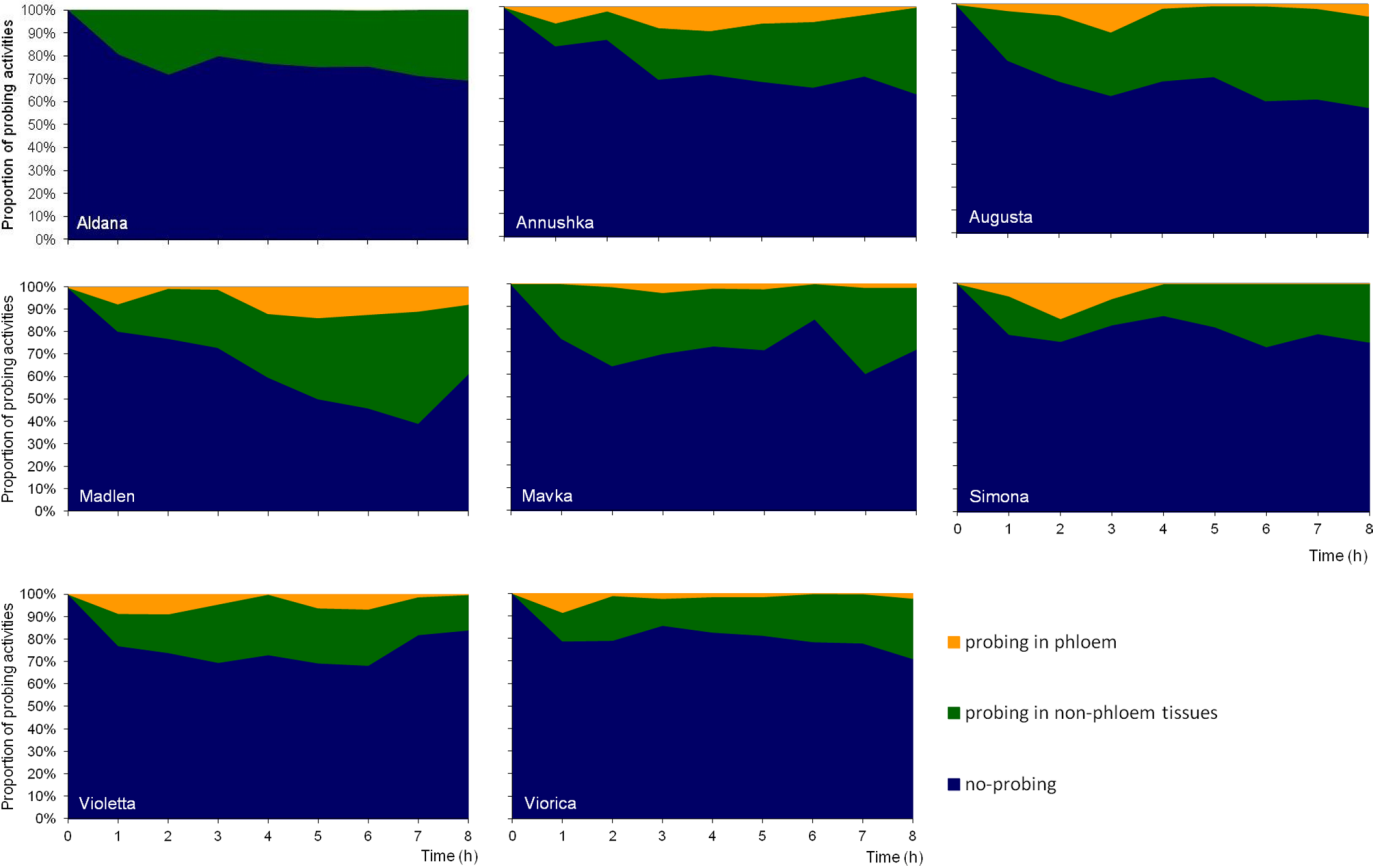
Temporal changes in probing behavior of *Acyrthosiphon pisum* on *Glycine max* cultivars according to the EPG monitoring.

**Figure 4:**
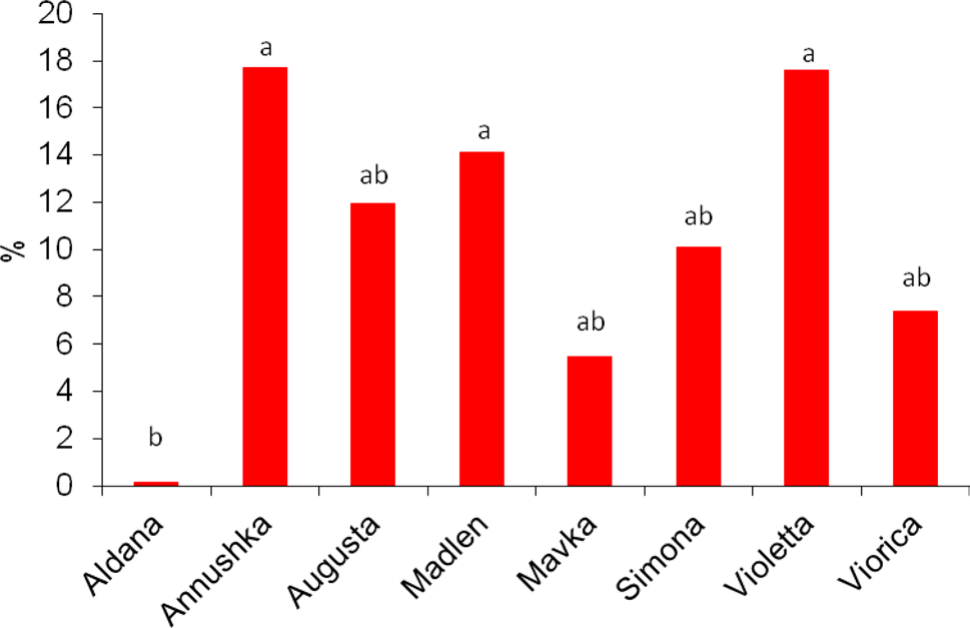
Frequency of phloem phase expressed as the proportion of *Acyrthosiphon pisum* that reached phloem sieve elements during 8-h access to *Glycine max* cultivars according to the EPG monitoring of aphid probing. Bars followed by the different letters are significantly different (LSD_0.05_: 0.337; ANOVA *F:* 1.41).

**Figure 5:**
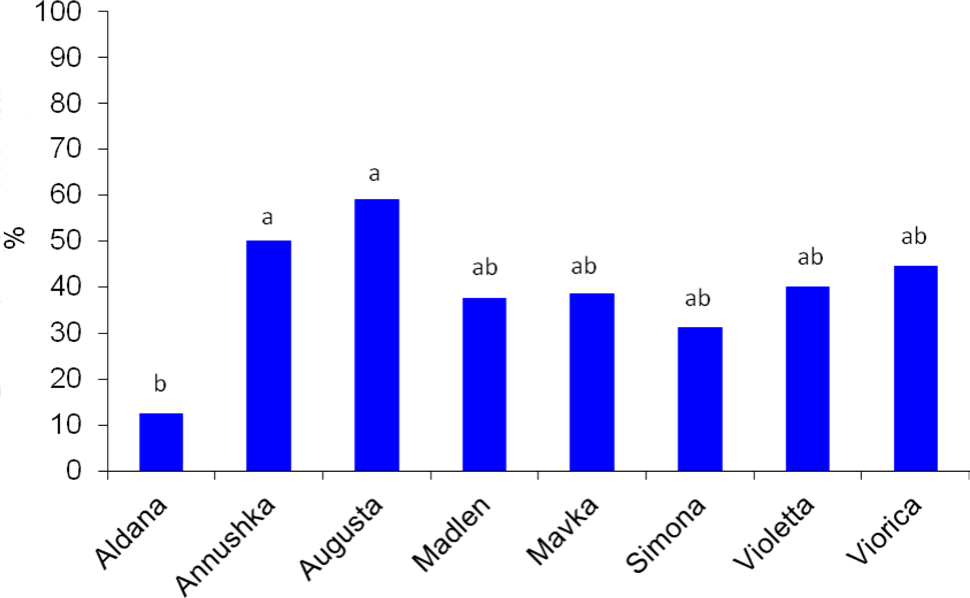
Proportion of phloem phase in all probing activities of *Acyrthosiphon pisum* on *Glycine max* cultivars recorded during the 8-h EPG monitoring of aphid probing, according to the formula: E/(C + E + G) × 100%, where C = pathway phase, E = phloem phase, G = xylem phase. Bars followed by the different letters are significantly different (LSD_0.05_: 13.84; ANOVA *F:* 1.46).

The 8-h EPG monitoring of pea aphid probing on soybean cultivars revealed activities defined as no-probing (= aphid stylets outside plant tissues), pathway phase (= aphid stylets in epidermis and mesophyll), xylem phase (= aphid stylets in xylem vessels), and phloem phase (= aphid stylets in sieve elements) (Table 1). Generally, no-probing predominated over pathway, xylem, and phloem phases on all soybean cultivars during the entire monitoring period (Fig. 3). The highest proportion of no-probing occurred in aphids on ‘Viorica’, ‘Simona’, ‘Aldana’, and ‘Violetta’ (80%, 78%, 75%, and 75% of experimental time, respectively) and the lowest on ‘Madlen’ (61%), while on the remaining cultivars, the proportion of no-probing ranged from 63% on ‘Augusta’ to 71% on ‘Annushka’ and ‘Mavka’ (Table 1, Fig. 3). The activities in non-phloem tissues were divided into pathway stylet penetration in epidermis and mesophyll and sap ingestion from xylem vessels. In mesophyll, short periods of derailed stylet movements (waveform ‘F’) occurred occasionally, irrespective of soybean cultivar. Therefore, in the present analysis all events of ‘F’ were included in the pathway stylet activities. The longest duration of xylem phase occurred on ‘Madlen’ (0.9 ± 1.1 h) and shortest on ‘Viorica’ (0.1 ± 0.3 h). The mean number of probes, the mean duration of probes, and the duration of the first probe were similar in all aphids on all soybean cultivars (Table 1). The shortest time to reach phloem phase from the onset of probing occurred in aphids on ‘Annushka’, and ‘Augusta’ and ‘Violetta’ (4.6 ± 3.4 and 5.1 ± 3.1, and 5.1 ± 3.0 h, respectively) (Table 1). However, on all cultivars except ‘Aldana’ and ‘Mavka’, there were individuals that were able to reach phloem phase as early as during the first hour after access to plants (Fig. 3). The overall success rate in reaching phloem vessels was highest on ‘Augusta’ and ‘Annushka’ (60% and 50% aphids showed phloem phase) and lowest on ‘Aldana’ (12.5%). On ‘Madlen’, ‘Mavka’, ‘Simona’, ‘Violetta’ and ‘Viorica’, similar proportion of aphids reached phloem phase (from 31% on ‘Simona’ to 44% on ‘Viorica’) (Fig. 4). As a result, the proportion of phloem phase in the studied pea aphid population was lowest on ‘Aldana’ (0.12%) and highest on ‘Annushka, Violetta, and Madlen (17.7%, 17.6%, and 14.1%, respectively) (Fig. 5). The number of probes that included the phloem phase was highest in aphids on ‘Augusta’ (1.0 ± 1.1) and lowest on ‘Aldana’ (0.1 ± 0.3) (Table 1). The frequency of phloem phase was low in all aphids on all soybean cultivars. The number of phloem phases per aphid ranged from 0.1 ± 0.3 on ‘Aldana’ to 1.3 ± 1.4 on ‘Augusta’. While on ‘Aldana’ the phloem phase included only the activity associated with salivation into sieve elements, on the remaining soybean cultivars the phloem phase consisted of both salivation and ingestion activities, including the periods of ingestion longer than 10 min (Table 1).

**Table 1:** Probing behavior of *Acyrthosiphon pisum* on *Glycine max* cultivars according to the 8-h EPG monitoring.

EPG trait/soybean cultivar	Aldana	Annushka	Augusta	Madlen	Mavka	Simona	Violetta	Viorica	LSD_0.05_
Total duration of non-probing (h)	6.0 ± 1.1 a (16/16)	5.7 ± 0.9 abc (16/16)	5.1 ± 1.7 bc (22/22)	4. 9 ± 1.8 c (16/16)	5.7 ± 1.4 abc (13/13)	6.2 ± 1.1 a (16/16)	6.0 ± 1.0 ab (15/15)	6.4 ± 1.2 a (18/18)	3316.7
Total duration of pathway phase C (h)	1.6 ± 0.9 ab (16/16)	1.3 ± 0.7 b (16/16)	2.3 ± 1.6 a (22/22)	1.6 ± 0.8 b (16/16)	1.8 ± 1.0 ab (13/13)	1.2 ± 0.8 b (16/16)	1.4 ± 0.8 b (15/15)	1.3 ± 0.9 b (18/18)	2505.6
Total duration of xylem sap ingestion G (h)	0.4 ± 0.4 bc (16/10)	0.6 ± 0.8 ab (16/11)	0.3 ± 0.4 bc (22/11)	0.9 ± 1.1 a (16/10)	0.4 ± 0.6 bc (13/7)	0.3 ± 0.4 bc (16/7)	0.2 ± 0.6 bc (15/3)	0.1 ± 0.3 c (18/5)	1520.4
Total duration of phloem phase E1 + E2 (h)	0.0 ± 0.0 b (16/2)	0.4 ± 0.8 ab (16/8)	0.3 ± 0.5 ab (22/13)	0.6 ± 1.3 a (16/6)	0.1 ± 0.2 b (13/5)	0.3 ± 0.5 ab (16/5)	0.4 ± 0.6 ab (15/6)	0.2 ± 0.3 ab (18/8)	1606.5
Number of probes	30.1 ± 14.4 ab (16/16)	26.9 ± 14.7 b (16/16)	39.1 ± 17.1 a (22/22)	32.5 ± 14.1 ab (16/16)	27.8 ± 11.9 b (13/13)	27.8 ± 11.6 b (16/16)	25.1 ± 12.8 b (15/15)	29.4 ± 13.2 ab (18/18)	9.73
Mean duration of probes (min)	4.5 ± 3.1 a (16/16)	8.1 ± 9.7 a (16/16)	7.0 ± 10.1 a (22/22)	7.6 ± 6.2 a (16/16)	5.5 ± 4.1 a (13/13)	5.0 ± 6.2 a (16/16)	7.4 ± 7.8 a (16/16)	3.6 ± 2.7 a (18/18)	290
Duration of the first probe (min)	3.7 ± 6.4 a (16/16)	5.7 ± 11.6 a (16/16)	1.1 ± 1.2 a (22/22)	0.4 ± 0.3 a (16/16)	3.4 ± 6.6 a (13/13)	0.6 ± 0.8 a (16/16)	6.9 ± 20.2 a (15/15)	3.7 ± 11.8 a (18/18)	395.6
Time from the first probe to the first phloem phase (h)	7.3 ± 1.1 a (16/2)	4.6 ± 3.4 (16/8)	5.1 ± 3.1 b (22/13)	5.7 ± 3.0 ab (16/6)	6.1 ± 2.5 ab (13/5)	5.7 ± 3.4 ab (16/5)	5.1 ± 3.0 b (15/6)	5.1 ± 3.2 b (18/8)	7325.8
Number of probes with phloem phase E	0.1 ± 0.3 c (16/2)	0.9 ± 1.3 ab (16/8)	1.0 ± 1.1 a (22/13)	0.8 ± 1.3 abc (16/6)	0.6 ± 1.0 abc (13/5)	0.3 ± 0.5 bc (16/5)	0.5 ± 0.7 abc (15/6)	0.8 ± 1.3 abc (18/8)	0.7166
Number of phloem phases E1 and E1 + E2	0.1 ± 0.3 c (16/2)	1.1 ± 0.3 ab (16/8)	1.3 ± 1.4 a (22/13)	1.1 ± 1.4 ab (16/6)	0.7 ± 1.8 abc (13/5)	0.3 ± 1.0 bc (16/5)	0.5 ± 0.5 abc (15/6)	0.9 ± 0.7 abc (18/8)	0.834
Number of phloem salivation phases E1	0.1 ± 0.3 c (16/2)	1.1 ± 1.4 ab (16/8)	1.1 ± 1.4 a (22/13)	1.1 ± 1.4 ab (16/6)	0.7 ± 1.8 abc (13/5)	0.2 ± 1.0 bc (16/5)	0.5 ± 0.5 abc (15/6)	0.9 ± 0.7 abc (18/8)	0.834
Number of phloem sap ingestion phases E2	0.0 ± 0.0 b (16/0)	0.8 ± 1.1 a (16/7)	0.6 ± 1.0 a (22/8)	0.5 ± 1.0 ab (16/4)	0.2 ± 0.4 ab (13/3)	0.3 ± 0.4 ab (16/4)	0.5 ± 0.7 ab (15/5)	0.5 ± 0.9 ab (18/6)	0.5416
Number of sustained phloem sap ingestion periods E2 > 10 min	0.0 ± 0.0 b (16/0)	0.5 ± 0.7 a (16/6)	0.4 ± 0.8 ab (22/5)	0.5 ± 1.0 a (16/4)	0.2 ± 0.4 ab (13/2)	0.3 ± 0.4 ab (16/4)	0.5 ± 0.7 a (15/5)	0.4 ± 0.7 ab (18/5)	0.4645

In brackets: number of aphids analyzed (= number of replications)/number of individuals performing the specific probing phase. In statistical analysis (Kruskal–Wallis test) all individuals were included; if no phloem phase occurred, the time from the 1st probe until the end of the recording was used; if a given probing event had not been recorded for an individual, the duration or the number of those events were given the values of 0. Different letters in rows show significant differences among cultivars (p < 0.05).

### Flavonoids in leaves of soybean cultivars

**Figure 6:**
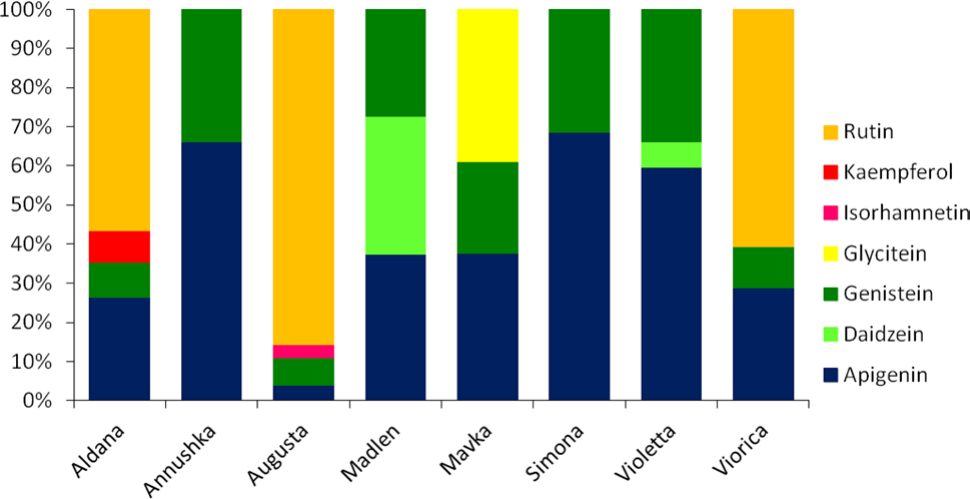
Proportion of flavonoids analyzed in *Glycine max* cultivars.

The total amount of flavonoids analyzed in leaves of soybean cultivars ranged from 1.80 ± 0.21 μg/g dry weight (d.w.) in ‘Annushka’ to 26.14 ± 1.94 μg/g d.w. in ‘Augusta’ (Table 2). These flavonoids included apigenin, daidzein, genistein, glycitein, isorhamnetin, kaempferol, and rutin, which occurred in different amounts and proportions in individual cultivars (Fig. 5). Apigenin and genistein occurred in all cultivars, daidzein occurred in ‘Madlen’ and ‘Violetta’, glycitein occurred in ‘Mavka’, isorhamnetin was detected in ’Augusta’, kaempferol was found in ‘Aldana’ and rutin occurred in ’Aldana’, ‘Augusta’, and ‘Viorica’ (Table 2, Fig. 6).

**Table 2:** Flavonoids analyzed in the leaves of *Glycine max* cultivars (μg/g dry weight).

Flavonoid/Cultivar	Aldana	Annushka	Augusta	Madlen	Mavka	Simona	Violetta	Viorica
Apigenin	2.43 ± 0.08 b	1.19 ± 0.33 c	1.05 ± 0.3 c	1.45 ± 0.22 c	1.07 ± 0.14 c	1.39 ± 0.22 c	5.38 ± 0.47 a	2.42 ± 0.36 b
Daidzein	n.d.	n.d.	n.d.	1.37 ± 0.01	n.d.	n.d.	0.5955 ± 0.0125	n.d.
Genistein	0.83 ± 0.07 cd	0.61 ± 0.19 d	1.78 ± 0.34 b	1.06 ± 0.01 c	0.68 ± 0.05 d	0.64 ± 0.05 d	3.05 ± 0.21 a	0.87 ± 0.03 cd
Glycitein	n.d.	n.d.	n.d.	n.d.	1.11 ± 0.03	n.d.	n.d.	n.d.
Isorhamnetin	n.d.	n.d.	0.89 ± 0.08	n.d.	n.d.	n.d.	n.d.	n.d.
Kaempferol	0.72 ± 0.06	n.d.	n.d.	n.d.	n.d.	n.d.	n.d.	n.d.
Rutin	5.20 ± 0.25	n.d.	22.42 ± 1.67	n.d.	n.d.	n.d.	n.d.	5.07 ± 0.76
Total	9.18 ± 0.24 b	1.80 ± 0.51 d	26.14 ± 1.94 a	3.88 ± 0.27c	2.85 ± 0.14 cd	2.03 ± 0.28 cd	9.03 ± 0.24 b	8.36 ± 1.41 b

n.d. = not detected; different letters in rows show significant differences among cultivars (apigenin: LSD_0.05_ = 0.622, *F* = 58.50; genistein: LSD_0.05_ = 0.369, *F* = 55.36; total flavonoids: LSD_0.05_ = 2.045, *F* = 163.2).

### Correlation analysis

Correlation analysis revealed significant positive correlation between: the total duration of phloem phase and the proportion of phloem phase in total probing (r = 0.854), the number of probes and the total flavonoids (r = 0.790), time from first probe to first phloem phase and the number of probes before first phloem phase (r = 0.753), the total duration of non-probing before first phloem phase and the number of probes before first phloem phase (r = 0.850) as well as apigenin and genistein (r = 0.792). A negative correlation coefficient was observed for the proportion of phloem phase in total probing and time from first probe to first phloem phase (r = − 0.818).

**Figure 7:**
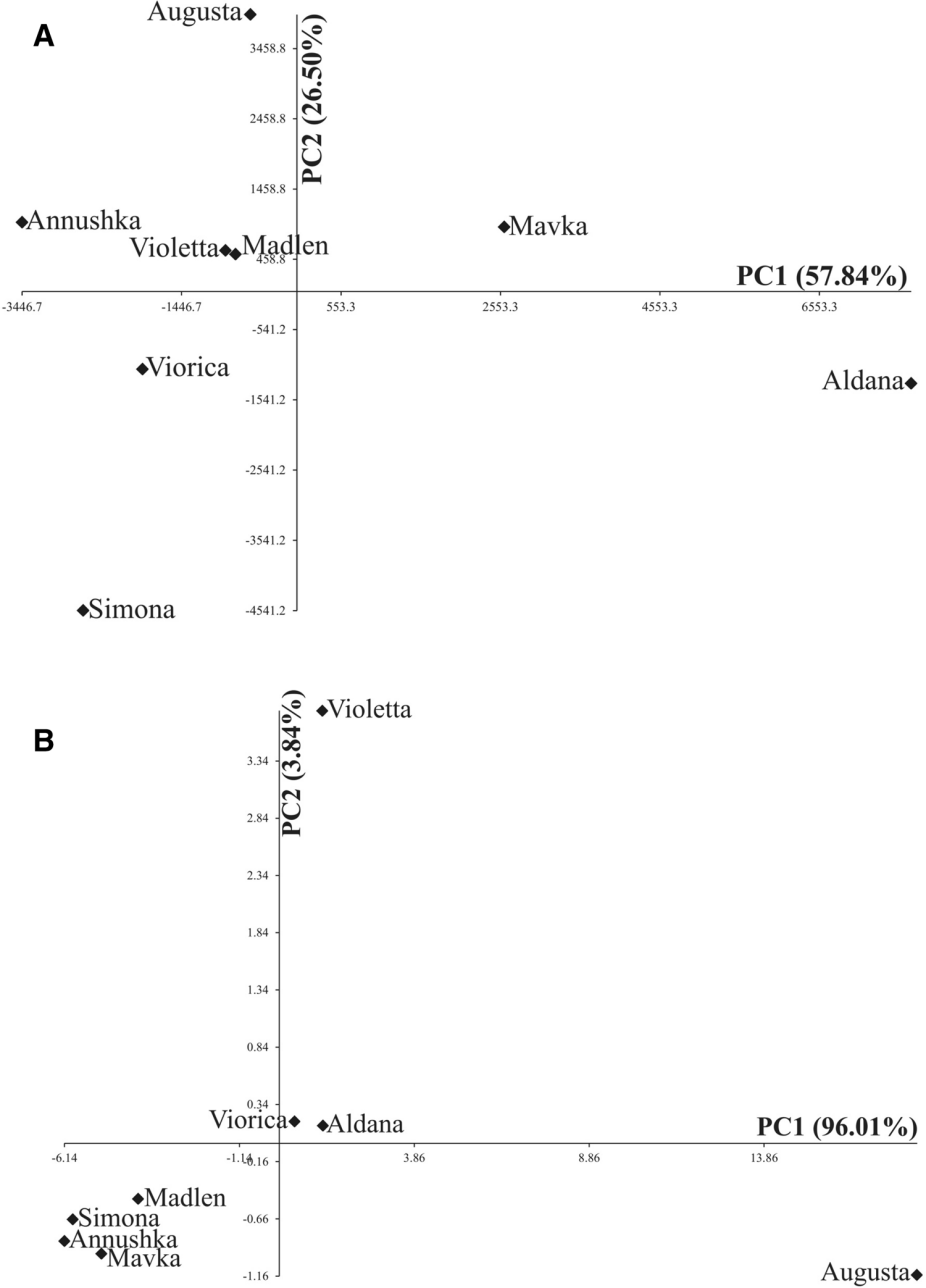
(**A**) Spatial distribution of eight *Glycine max* cultivars in terms of the first two principal components of nine observed traits of EPG-monitored probing behavior of *Acyrthosiphon pisum*. (**B**) Spatial distribution of eight *Glycine max* cultivars in terms of the first two principal components of three flavonoid traits.

Distribution of soybean cultivars in terms of the first two principal components of nine observed traits of EPG is presented in Fig. 7A. The first two principal components accounted for 84.34% of total multivariate variability between studied cultivars. Distribution of eight soybean cultivars in terms of the first two principal components of three flavonoids traits is presented in Fig. 7B. The first two principal components accounted for 99.85% of total multivariate variability between eight soybean cultivars.

## Discussion

Generally, *A. pisum* was willing to probe into leaf tissues of all soybean cultivars studied presently. However, the probes were usually terminated within less than four to seven minutes. In consequence, no-probing was the main activity of the pea aphid on soybeans and the success rate in finding the sieve elements was very low or aphids did not reach phloem phase at all within the 8-h period of access to plants. In those rare cases when the pea aphid did find phloem vessels, the phloem phase was very short. The new-born nymphs of the pea aphid did not survive beyond one or two days on all soybean cultivars studied. In the free-choice test, aphids avoided all soybean cultivars in favor of *P. sativum.* To understand and provide the possible explanation of the pea aphid behavior on soybeans, the analysis of behavioral events that lead to host-plant selection and acceptance must be considered. There are two major phases in this process: (i) the host-plant location in the environment and (ii) the examination of plant features once the prospective host-plant has been traced and approached^44^. In our study, we concentrated upon the latter phase that comprises the assessment of plant internal traits, mainly of biochemical nature^27,45^. In contrast to herbivore insects with biting-chewing mouthparts that are equipped with external contact taste receptors, aphids possess sucking-piercing mouthparts that lack such sensory elements^27^. The essential taste organ is located in the pharynx^46^, therefore aphids need to take samples of plant sap during probing with their stylets to assess the suitability of the potential host plant^27^. Consequently, aphid probing behavior reflects the susceptibility of plants to aphid infestation on the one hand and aphid ability to overcome plant defenses on the other^27^. Generally, aphids respond to the quality of plant sap by either continuing or terminating stylet penetration^47,48^. The time required by aphid stylets to pass one layer of cells is approximately two to three minutes^49^. Stylet withdrawal by aphids after a short probe in the outer leaf tissues suggests the presence of probing deterrents in these tissues. This is often observed in incompatible plant-aphid associations^23^, on resistant plant cultivars^48^, or when the non-acceptable xenobiotics are applied to aphid host-plants^50^. The soybean genotypes studied evoked a spectrum of behavioral responses from *A. pisum*. We demonstrated that the pea aphids withdrew their stylets four to seven minutes after the beginning of a probe, which means that the probing aphids must have encountered the deterrent factors either in the first (epidermis) or second/third (mesophyll) tissue layer in soybean leaves. This was especially noticeable in the cultivar ‘Aldana’, on which the proportion of no-probing time in relation to other aphid activities was highest as compared to other soybean cultivars studied. The mean duration of a probe was approximately four minutes and almost all aphids on this cultivar failed to reach phloem vessels and commence feeding during eight hours of access to the plants, which means that the probes included chiefly stylet activities in non-phloem tissues and points at the strong activity of antixenosis factors in the outer leaf tissues. The described characteristic behavior was also typical of *A. pisum* probing on resistant lupine cultivars and unpalatable species of forage and grain legumes, on which non-probing activities prevailed over stylet penetration, the probes were terminated usually 3–5 min after stylet insertion in plant tissues, and the phloem phase was short or did not occur^24–26,48^. Low number of probes before the first phloem phase, short duration of no-probing, relatively short time to reach phloem phase, and sap ingestion sustained over many hours with no interruption indicate that little or no antixenosis factors are present in tissues encountered before reaching sieve elements and in the phloem vessels by *A. pisum* on susceptible legumes^24–26,48^. The predominance of no-probing activities occurred also on soybean cultivars ‘Annushka’, ‘Augusta’, ‘Madlen’, ‘Mavka’, ‘Simona’, ‘Violetta’, and ‘Viorica’. However, aphids on these cultivars were able to reach phloem and commence feeding. Among these cultivars, the frequency of phloem sap ingestion phase on ‘Annushka’ and ‘Augusta’ was higher than in ‘Madlen’, ‘Mavka’, ‘Simona’, ‘Violetta’, and ‘Viorica’ while the total duration of phloem phase was highest in ‘Madlen’. The reduced duration of phloem sap uptake results in the impediment of aphid survival and development, which happened to the cabbage aphid *Brevicoryne brassicae* L. on resistant rapeseed cultivars^51^. Among soybean cultivars studied, the lowest survival of newborn nymphs was on ‘Aldana’ and highest on ‘Madlen’.

Aphids on all soybean cultivars showed stylet activities associated with the ingestion of sap from xylem vessels. Generally, the mean duration of xylem phase was comparable to the duration of the phloem phase. However, on ‘Aldana’ the xylem phase was the key activity associated with ingestion from vascular tissues, as the phloem phase was practically absent on this cultivar. Water uptake by aphids is generally considered as an osmoregulatory strategy in response to phloem sap dietary osmotic pressure and dehydration caused by drought^52^. On the other hand, it has been proposed that xylem sap ingestion is initiated to reduce the negative impact of plant toxins^53^, which may also have been the case in the present study.

Plant allelochemical antixenosis against aphids is based mainly on bioactive compounds, such as hydroxamic acids, alkaloids, polyphenols, flavonoids, terpenoids or saponins^54^. In our study, we concentrated on flavonoids which are well known for their detrimental effect on insect herbivores including the pea aphid^55–58^. The correlation analysis revealed that the total amount of the group of flavonoids analyzed did not affect the pea aphid probing behavior significantly. It was especially noticeable in cultivar ‘Augusta’, on which the aphid feeding success was relatively high despite the highest observed content of the analyzed flavonoids of all soybean cultivars studied. Apigenin and genistein were detected in all cultivars. Both apigenin and genistein are known for their anti-herbivore properties. Apigenin is highly toxic to larvae of southern house mosquito *Culex quinquefasciatus* Say (Diptera: Culicidae), affects the fecundity, mortality, and food consumption of Formosan termite *Coptotermes formosanus* Shiraki (Blattodea: Isoptera: Rhinotermitidae), shows antifeedant activity against striped flea beetles *Phyllotreta striolata* (Coleoptera: Chrysomelidae)^59–61^. Apigenin reduced the pea aphid abundance and phloem sap ingestion on alfalfa, caused a reduction in the number and duration of probes when added to saponin mixtures in artificial diets, and was accumulated in vegetative parts of aphid-infested pea plants^62^. In soybean, genistein had negative effects on the behavior and biology of *Anticarsia gemmatalis* Hübner (Lepidoptera: Noctuidae), *Piezodorus guildinii* (Hemiptera: Pentatomidae), and *Trichoplusia ni* (Hübner) (Lepidoptera: Noctuidae)^63–65^. The application of genistein in artificial diet decreased the feeding efficiency and reduced the survival rate of *A. pisum* and the increases in genistein conferred resistance against the pea aphid in *M. sativa*^57,66^. Considering the presence of both genistein and apigenin in all soybean cultivars studied it is reasonable to infer that these flavonoids are responsible for the general negative response of the pea aphid to *G. max*. The subtle differences observed in the acceptability of soybean cultivars may be due to the content of daidzein, glycitein, isorhamnetin, kaempferol and rutin, which were identified in individual cultivars. However, these flavonoids differ in their role in constitutive and induced plant resistance against biotic stressors. Daidzein and genistein are associated with the observed antibiosis resistance of soybeans to the soybean aphid, and are induced in soybean leaves by the feeding of *Spodoptera litura* (L.) (Lepidoptera: Noctuidae) and *A. gemmatalis*^67–69^. In the present study, daidzein was found in the only one relatively acceptable to the pea aphid cultivar ‘Madlen’ and relatively non-accepted ‘Violetta’. Glycitein is induced by the feeding of *A. gemmatalis* but not by *S. litura*^67^. In our research, we detected glycitein in the relatively non-accepted cultivar ‘Mavka’. Isorhamnetin is associated with the resistance of cowpea *Vigna unguiculata* L. Walp. against *Aphis fabae* (Scop.) and has promising potential as an anthelmintic against *Haemonchus contortus* (Rudolphi, 1803) Cobb (Nematoda: Trichostrongylidae)^69,70^. In our study, isorhamnetin was found in the relatively acceptable cultivar ‘Augusta’. Kaempferol occurs in higher quantity in cowpea cultivars resistant to cowpea aphid as compared to susceptible cultivars^69^. An increase in the content of kaempferol was observed due to the feeding of *A. gemmatalis* on soybeans^71^. The level of kaempferol in broccoli did not change due to herbivory of *B. brassicae* and *M. persicae*^72^ and did not correlate with the number of *A. pisum* colonizing *P. sativum* seedlings^38^. In our study, kaempferol was detected only in the cultivar ‘Aldana’ that was the least acceptable soybean cultivar to *A. pisum* in the present study. Rutin is generally considered as associated with plant resistance against herbivores^73^. High concentration of rutin was found in soybean cultivars resistant to *A. gemmatalis* and *P. guildinii*^63,64^. Rutin is toxic to the woolly apple aphid *Eriosoma lanigerum* (Hausmann)^73,74^. In our study, rutin occurred in cultivars ‘Aldana’, ‘Augusta’, and ‘Viorica’, which differed in their susceptibility to *A. pisum*, ‘Aldana’ being relatively least acceptable and ‘Augusta’ relatively most acceptable cultivar to the pea aphid.

Aphid stylets penetrate plant tissues mostly within the apoplast, between the cellulose and hemicellulose fibres of the secondary cell walls and their work is mostly mechanical with still not well known support of salivary enzymes^27,75–77^. Mechanical problems with the stylets, visualized in EPG recordings as waveform ‘F’ during mesophyll phase of probing have not been well defined, yet^77,78^. Various studies reported that the derailed mechanics may occur in aphids on resistant plants^79^ and in aposymbiotic aphids^80^, may depend on the age of plants^79^ or may be a function of insect age and plant resistance level^53^. The incidence of ‘F’ in our study was negligible, which allows us to conclude that it is likely that no mechanical obstacles occur in leaf tissues of soybean cultivars studied. Thus, biophysical antixenosis was not the reason of the rejection of these cultivars by *A. pisum* biotype ‘G’.

The statistical analysis (PCA) performed independently for aphid probing and plant chemistry showed no similarities in the groupings of soybean cultivars studied. Therefore, no unequivocal classification of cultivars that would have included all analyzed traits was possible. Nevertheless, taking into account the pea aphid probing behavior and relative feeding success as well as the survival, the soybean cultivars studied can be categorized according to *A. pisum* preferences and the assumed backgrounds of these preferences into four groups. Group I—relatively susceptible—cultivar ‘Madlen’, on which the pea aphid feeding success and survival were highest. Group II—medium susceptible—‘Annushka’ and ‘Augusta’, on which the feeding success and survival were lower than in ‘Madlen’ but higher than in ‘Mavka’, ‘Simona’, ‘Violetta’, and ‘Viorica’. Group III—medium resistant—‘Mavka’, ‘Simona’, ‘Violetta’, and ‘Viorica’, on which the feeding success and survival were lower than in ‘Annushka’ and ‘Augusta’ but higher than in ‘Aldana’. Group IV—highly resistant—‘Aldana’ on which the pea aphid feeding success and the survival were lowest.

In conclusion, we have confirmed that soybean is a relatively unsuitable host for the pea aphid, which we have cautiously determined in our previous studies^26^. On all soybean cultivars, *A. pisum* readily probed into leaf tissues but the probes were usually terminated before reaching vascular tissues. In consequence, the phloem phase was significantly delayed or did not occur, the ingestion of phloem sap was limited or prevented, and aphid survival was dramatically impeded. Thus, we can infer the existence of antixenosis factors in peripheral leaf tissues of all soybean cultivars studied. Nevertheless, as stylet penetration of *A. pisum* in peripheral and vascular tissues was not entirely impeded on any cultivar of soybeans, *G. max* may be considered a possible source of semi-persistent and persistent viruses, respectively, that may be acquired by the pea aphid and transferred to other legumes and vice versa. Antixenosis in soybean cultivars studied is primarily of biochemical nature. The potency of antixenosis factors differs among soybean cultivars, which was reflected in differences in the acceptance of these cultivars by *A. pisum.* In our opinion, the spectrum and not the amount of flavonoids in soybean leaves was responsible for the varying pea aphid response to individual cultivars. The content of apigenin and genistein in all soybean cultivars studied probably made all of them relatively unacceptable to *A. pisum*. We hypothesize that kaempferol in ‘Aldana’ might be responsible for the observed strong antixenosis resistance of this cultivar to *A. pisum.* However, the impact of individual soybean flavonoids needs a further study. There was no knowledge on the background of susceptibility or resistance of soybean cultivars to *A. pisum* infestation prior to our study. The results of our survey provide the first detailed data that can be used for reference studies in the future.

## Material and methods

### Plants and aphids

Eight cultivars of genetically unmodified soybeans were studied: ‘Aldana’, ‘Annushka’, ‘Augusta’, ‘Madlen’, ‘Mavka’, ‘Simona’, ‘Violetta’, and ‘Viorica’. These cultivars were selected because they represent various regions of origin in Eastern and Central Europe: Bulgaria (‘Simona’), Lithuania (‘Violetta’), Poland (‘Aldana’, ‘Augusta’, ‘Madlen’, ‘Mavka’), Romania (‘Viorica’), and Ukraine (‘Annushka’)^81^, and belong to different maturity groups in these regions: ‘Annushka’ and ‘Augusta’ are very early maturing cultivars, ‘Aldana’, ‘Simona’, ‘Violetta’, ‘Viorica’—early, ‘Mavka’—semi-early, and ‘Madlen’—late maturing cultivars^82^. The seeds were provided by Hodowla Soi Agroyoumis Sp. z o. o. (Kordeckiego 20, 37-420 Rudnik nad Sanem, Poland). Plants were grown in commercial soil in 9 cm diam. plastic pots, in the chamber Sanyo MLR-351H (Sanyo Electronics Co. Ltd.) at 20 °C, 65% r.h., and L16:8D photoperiod. The plants were watered regularly and no fertilizers were applied.

The laboratory culture of *P. sativum-*derived *Acyrthosiphon pisum* (biotype ‘G’ according to^19^) was maintained on *Pisum sativum* cv. ‘Milwa’ in the laboratory at 20 °C, 65% r.h., and L16:8D photoperiod. The seeds of *P. sativum* were purchased from HR Smolice Sp. z o. o. Grupa IHAR (Oddział Przebędowo, 62–095 Murowana Goślina, Poland).

The study did not involve the collection of plants and insects in nature. Plants used in the present study were grown from commercially available seeds. Insects used in the present study were collected from the laboratory culture kept at the Department of Botany and Ecology, University of Zielona Gora, Poland since 2000. The research complies with relevant institutional, national, and international guidelines and legislation.

### Free-choice test

To evaluate the antixenosis resistance in soybean cultivars, we performed free-choice tests. *Pisum sativum* cv. ‘Milwa’ was used as susceptible check for all soybean cultivars. Plants for the tests were cultured as described. Shoots of plants at 14 BBCH growth stage (trifoliate leaf on the 4th node unfolded)^83^ were excised. The cut end of each shoot was covered with moist cotton wool and placed in an eppendorf vial. The prepared shoots were placed randomly at equidistance from each other in a circular manner on the bottom of a glass vial (240 mm diam, 10 mm high) and 50 apterous females of *A. pisum* were introduced in the centre of the arena. The vial was covered with a gauze, transferred to the growing chamber Sanyo MLR-350 H (Sanyo Electronics Co. Ltd.) and kept there at 21 ± 1 °C, 65% r.h., and L16:8D photoperiod. The number of aphids on each plant shoot was counted 24 h later. The experiment was replicated 15 times.

### Survival tests

One adult apterous female of *A. pisum* was placed on a plant at 14 BBCH growth stage (trifoliate leaf on the 4th node unfolded)^83^ for 24 h. After 24 h, the female and all progeny except one nymph were removed. Each plant was isolated within a plastic cylinder with a fine mesh on top. The development of the nymph was monitored daily. The experiment was replicated 15 times for each soybean cultivar. The tests were conducted in an environmental chamber Sanyo MLR-351H (Sanyo Electronics Co. Ltd.) at L16:D8 photoperiod, 21 ± 1 °C, and 70% r.h.

### Aphid probing behavior

The probing behavior of *A. pisum* was monitored using the technique of electronic registration of aphid probing in plant tissues, known as Electrical Penetration Graph or electropenetrography (EPG)^27^. Aphid and plant were made parts of an electric circuit, which was completed when the aphid inserted its stylets into the plant. Weak voltage was supplied in the circuit, and all changing electric properties were recorded as EPG waveforms that could be correlated with aphid activities and stylet position in plant tissues^27^. In the present study, adult apterous aphids were attached to a golden wire electrode with conductive silver paint and starved for 1 h prior to the experiment. Probing behavior of *A. pisum* on soybean cultivars was monitored for 8 h continuously with 4- and 8-channel DC EPG recording equipment. Signals were saved on the computer and analyzed using the PROBE 3.1 software provided by W. F. Tjallingii (www.epgsystems.eu). The following aphid behaviors were distinguished: no penetration (waveform ‘np’—aphid stylets outside the plant), pathway phase—penetration of non-phloem tissues (waveforms ‘ABC’), derailed stylet movements (waveform ‘F’), salivation into sieve elements (waveform ‘E1’), ingestion of phloem sap (waveform ‘E2’), and ingestion of xylem sap (waveform ‘G’). The E1/E2 transition patterns were included in E2. Waveforms ‘F’ occurred sporadically, therefore these events were combined with pathway activities in all calculations and defined as non-phloem activities. The waveform patterns that were not terminated before the end of the experimental period (8 h) were included in the calculations. In sequential parameters, when time to waveforms related to phloem phase (E1 or E2) was calculated, the time from the 1st probe until the end of the recording was used if no phloem phase occurred. In non-sequential parameters, when a given waveform had not been recorded for an individual, the duration of that waveform was given the value of 0.

Aphids for EPG experiments were 2–3 days old (2–3 days after the final molt) viviparous apterous *A. pisum*. The plants of *G. max* used in the bioassays were at 14 BBCH growth stage (trifoliate leaf on the 4th node unfolded)^83^. Each aphid was given access to a freshly prepared plant. Each plant-aphid set was considered as a replication and was tested only once. The number of replications for each plant cultivar/aphid combination was 24. However, only the replications that included complete 8-h recording were kept for analysis, which were: ‘Aldana’, n = 16; ‘Annushka’, n = 16; ‘Augusta’, n = 22; ‘Madlen’, n = 16; ‘Mavka’, n = 13; ‘Simona’, n = 16; ‘Violetta’, n = 15, ‘Viorica’, n = 18. Recordings that terminated due to aphid falling from the plant or where EPG signal was unclear were discarded from analysis. All bioassays started at 10:00–11:00 h MEST (Middle European Summer Time). Aphids show distinct diurnal feeding activity, with peak activity during day time, independently of host plants^27,84,85^.

### High-performance liquid chromatography of flavonoids

The dried soybean leaves, of different botanical varieties, i.e., ‘Annushka’, ‘Aldana’, ‘Augusta’, ‘Madlen’, ‘Mavka’, ‘Simona’, ‘Violetta’ and ‘Viorica’ (1.2 g of each) were homogenized in an aqueous ethanol solution (80%) using a Diax 900 homogenizer. The resulting suspension was centrifuged (12,000 rpm, 10 min) and the supernatant solution was collected in a graduated flask and the pellet was reconditioned. This operation was repeated three times, and the obtained extracts were combined. The homogenization procedure in combination with the extraction was carried out in such a way that the final volume of the extract was 100 ml. From the prepared ethanol extracts, 10 ml was taken and evaporated to dryness in a rotary evaporator under reduced pressure at 40 °C. The dry extracts were dissolved in 100% methanol to a volume of 1 ml. Resulting methanolic extracts containing flavonoids compounds were analyzed by HPLC–ESI–MS/MS.

The content of: ampelopsin, apigenin, daidzein, genistein, glycitein, hesperetin, hesperidin, isorhamnetin, kaempferol, luteolin, naryngin, quercetin, rutin, taxifolin was determined. The selection of the flavonoid spectrum for analysis was based on literature data^86–89^.

Individual pure flavonoids were purchased from Sigma–Aldrich (Poland). Ethanol, HPLC gradient grade methanol and acetonitrile were supplied by Merck (Germany). Formic acid was purchased from Sigma-Aldrich (Poland). Stock standard solutions of individual flavonoids (50 mg/1) were prepared by dissolving appropriate amounts of solid reagents in methanol. Mixed working standard solutions of flavonoid compounds at 20, 10, 5, 2.5 and 1 mg/1 or lower concentrations were prepared by appropriate dilutions of stock standard solutions.

The chromatographic analysis was carried out with a Shimadzu LC system, comprising a LC20-AD binary pump, a DGU-20A5 degasser, a CTO-20AC column oven and a SIL-20AC autosampler, connected to a 3200 QTRAP hybrid triple quadrupole (Applied Biosystem, MDS SCIEX, USA) with electrospray ionization source (ESI) operated in negative-ion mode. Phenolic compounds were separated on a Phenomenex Luna C-18 column (100 × 2.0 mm × 3.0 µm) with a pre-column, both maintained at 30 °C. A 7.4 mmol/l solution of formic acid (pH 2.8, eluent A) and acetonitrile (eluent B) were used. The mobile phase was delivered at 0.2 ml/min in a linear gradient mode as follows: 0–2 min 10% B, 30 min 60% B, 40 min 100% B, 55 min 10% B. Flavonoids were identified by comparing their retention times and m/z values of precursor and resulting fragmentation product ions in their MS and MS/MS spectra, respectively, to those obtained for respective standard solutions analyzed under the same conditions. The quantification of flavonoids was done using calibration curves obtained in the SRM (single reaction mode) mode^90,91^.

### Statistical analysis

All statistical calculations were performed using StatSoft, Inc. (2014) STATISTICA (data analysis software system), version 12. Parameters of the free-choice test and aphid performance (nymph survival) were analyzed using Kruskal–Wallis test and post-hoc multiple comparisons of mean ranks for all groups (Dunn’s test). EPG parameters describing aphid probing behavior were calculated manually and individually for every aphid and the mean and standard errors were subsequently calculated using the EPG analysis Excel worksheet created by the authors especially for this study. All aphids were included in analysis. If the specific trait did not occur in the individual EPG recording, the value for this trait was given zero. Data thus obtained were analyzed by Kruskal–Wallis test and post-hoc multiple comparisons of mean ranks for all groups (Dunn’s test). Additionally, the relationships among all the traits were estimated on the basis of correlation coefficients. The graphic distribution of cultivars, described by means of the observed traits, was obtained by means of the principal components analysis (PCA). Correlation and PCA analyses were done in GenStat 18.
